# Functional phosphoproteomic mass spectrometry-based approaches

**DOI:** 10.1186/2001-1326-1-20

**Published:** 2012-09-05

**Authors:** Elena López, Xiangdong Wang, Luis Madero, Juan López-Pascual, Martin Latterich

**Affiliations:** 1 Hospital Universitario Niño Jesús, Av. Menéndez Pelayo 65, 28009, Madrid, Spain; 2 Clinical Sciences Lund, Skåne University Hospital and Lund University, Lund, SE-22185, Sweden; 3 Hospital Universitario 12 de Octubre, Av. De Córdoba s/n, 28041, Madrid, Spain; 4 Proteogenomics Research Institute for Systems Medicine, 11107 Roselle Street, San Diego, CA, 92121-1206, USA

**Keywords:** Phosphorylation, Signaling pathways, Phosphoproteomics, Mass spectrometry

## Abstract

Mass Spectrometry (MS)-based phosphoproteomics tools are crucial for understanding the structure and dynamics of signaling networks. Approaches such as affinity purification followed by MS have also been used to elucidate relevant biological questions in health and disease.

The study of proteomes and phosphoproteomes as linked systems, rather than research studies of individual proteins, are necessary to understand the functions of phosphorylated and un-phosphorylated proteins under spatial and temporal conditions. Phosphoproteome studies also facilitate drug target protein identification which may be clinically useful in the near future.

Here, we provide an overview of general principles of signaling pathways versus phosphorylation. Likewise, we detail chemical phosphoproteomic tools, including pros and cons with examples where these methods have been applied. In addition, basic clues of electrospray ionization and collision induced dissociation fragmentation are detailed in a simple manner for successful phosphoproteomic clinical studies.

## Background

Proteomics technologies are of great use for developing reference maps of targets of kinase inhibitors. This implies a difficult but promising issue, as it concerns the determination of the network-level response to different inhibitor treatments. The challenges and cautions associated with each method will be described in a simple manner.

With advances of techniques available, the next decade should see a rapid increase in our understanding of signaling network regulation. This, at the same time, implies more efficient therapeutic strategies.

Comparative proteomics can distinguish small but important changes in protein modifications in their structure, thus facilitating drug target protein identification.

In addition, phosphoproteomic approaches can be exploited to monitor changes in phosphorylation events in order to characterize drug actions on cell signaling pathways and/or signaling cascades. Likewise, functional proteomic and phosphoproteomic approaches can be used to: (i) improve the knowledge or the clarification of the mechanism of a drug action for a specific disease, (ii) achieve relevant protein-identification of disease related to a specific signaling network and (iii) reach the important step of innovation of novel drug targets for specific pathologies [1].

In spite of all these uses of proteomics and phosphoproteomics, there is limited clinical success of therapeutics targeting cellular signaling processes owing to the complex feedback regulation encoded within the signaling network. Signaling networks are controlled mainly by phosphorylation.

When a protein is phosphorylated, this implies that the protein structure contains a phosphate group. It should be assumed that the phosphate group assignments -during phosphoproteomic studies- in a low expressed phosphorylated protein (e.g. some kinases) imply difficulties such as: (a) the low abundance of those signaling molecules within cells, (b) the stress/stimulation time-duration, as only a small fraction of phosphorylated kinases are available at any given time as a result of a stimulus, (c) and, the time adaptation over signaling pathways is a relevant and fast factor for kinases phosphorylation. It should be taken into account that the current phosphoenrichment methods are mainly successful to purify phosphopeptides from highly expressed proteins [2].

**Figure 1:**
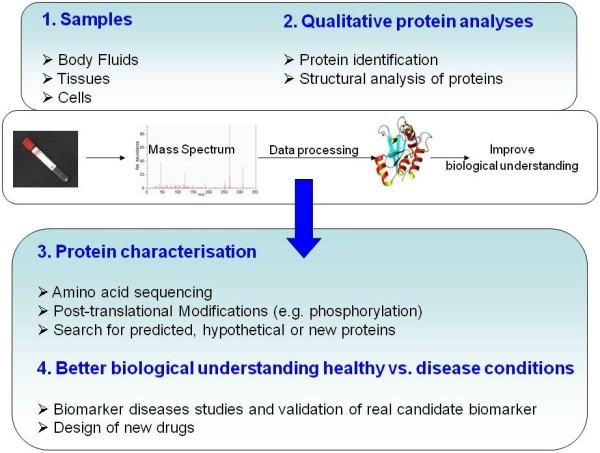
**A possible proteomic work-flow for clinical studies.** For identification, in-gel or in-solution proteins of interest are excised, washed and enzimatically digested to extract peptides for further analysis by MS. The mass to charge (m/z) ratio of the retained peptides can be measured by a mass spectrometer. Data-computer analysis is carried out which mainly includes: identification and classification of proteins, validation of identified protein, interpretation and classification of the protein lists, structural analysis of proteins, PTM analysis and Cellular composition.

Proteomics undoubtedly holds great promise for disease diagnosis, prognosis and prediction of therapeutic outcome on an individualized basis. However, proper study design is always necessary to optimize and establish the right proteomic strategy for each clinical sample to be studied. The resulting data must, in addition, be evaluated by scientists and clinicians. Figure 1 illustrates a possible proteomic work-flow useful for clinical studies.

## Importance of phosphoproteomics for signal transduction pathways

Protein phosphorylation events often play important roles in broad signaling networks within a cell. Phosphorylation of kinases frequently regulates their own activity, but they are commonly under-represented in phosphoproteomic studies. This is due, in part, to their low expression within the cell and in part due to the difficulty in detecting highly negatively charged peptides in the mass spectrometer.

Nevertheless, this drawback can be overcome though kinase affinity purification techniques that enrich for these low expressed proteins.

Phosphatases also play important roles in regulating signaling pathways through the removal of phosphate groups from proteins. In fact depleting cells of specific protein phosphatases and using phosphoproteomic approaches, can determine which proteins are regulated by the phosphatase of interest [2-7].

### General principles of signaling pathways versus phosphorylation

Phosphorylation is the addition of a phosphate (PO_4_^ 3-^) group to a protein or another organic molecule. Phosphorylation turns many protein enzymes “on” and “off”, thereby altering their function and activity. Protein phosphorylation in particular plays a significant role in a wide range of cellular processes. On the other hand, reversible phosphorylation of proteins is an important regulatory mechanism that occurs in both prokaryotic and eukaryotic organisms [8-14].

Within a protein, phosphorylation can occur on several amino acids. Phosphorylation on serine is the most common, followed by threonine. Tyrosine phosphorylation is relatively rare. However, since tyrosine phosphorylated proteins are relatively easy to purify using antibodies, tyrosine phosphorylation sites are relatively well understood. Histidine and aspartate phosphorylation occurs in prokaryotes as part of two-component signaling and in some cases in eukaryotes in some signal transduction pathways [15].

Kinases phosphorylate proteins and phosphatases dephosphorylate proteins. Many enzymes and receptors are switched "on" or "off" by phosphorylation and dephosphorylation. Reversible phosphorylation results in a conformational change in the structure of many enzymes and receptors, causing them to become activated or deactivated. Phosphorylation usually occurs on serine, threonine, tyrosine and histidine residues in eukaryotic proteins. Histidine phosphorylation of eukaryotic proteins appears to be much more frequent than tyrosine phosphorylation. In prokaryotic proteins phosphorylation occurs on the serine, threonine, tyrosine, histidine or arginine or lysine residues [16,17].

The addition of a phosphate (PO_4_) molecule to a polar group of an amino acid residue can turn a hydrophobic portion of a protein into a polar and extremely hydrophilic portion of molecule. Thus, it can introduce a conformational change in the structure of the protein via interaction with other hydrophobic and hydrophilic residues in the protein. Upon the deactivating signal, the protein becomes dephosphorylated again and stops working. This is the mechanism in many forms of signal transduction.

The regulatory roles of phosphorylation include [18-25]:

(a) Biological thermodynamics of energy-requiring reactions (for example, phosphorylation of Na^+^/K^+^-ATPase during the transport of sodium (Na^+^) and potassium (K^+^) ions across the cell membrane in osmoregulation to maintain homeostasis of the body's water content).

(b) Mediates enzyme inhibition (for example, phosphorylation of the enzyme GSK-3 by AKT (Protein kinase B) as part of the insulin signaling pathway)

(c) Protein-protein interaction via "recognition domains." (for example, phosphorylation of the cytosolic components of NADPH oxidase, a large membrane-bound, multi-protein enzyme present in phagocytic cells, plays an important role in the regulation of protein-protein interactions in the enzyme)

(d) Protein degradation - for example, in the late 1990s, it was recognized that phosphorylation of some proteins causes them to be degraded by the ATP-dependent ubiquitin/proteasome pathway. These target proteins become substrates for particular E3 ubiquitin ligases only when they are phosphorylated.

**Figure 2:**
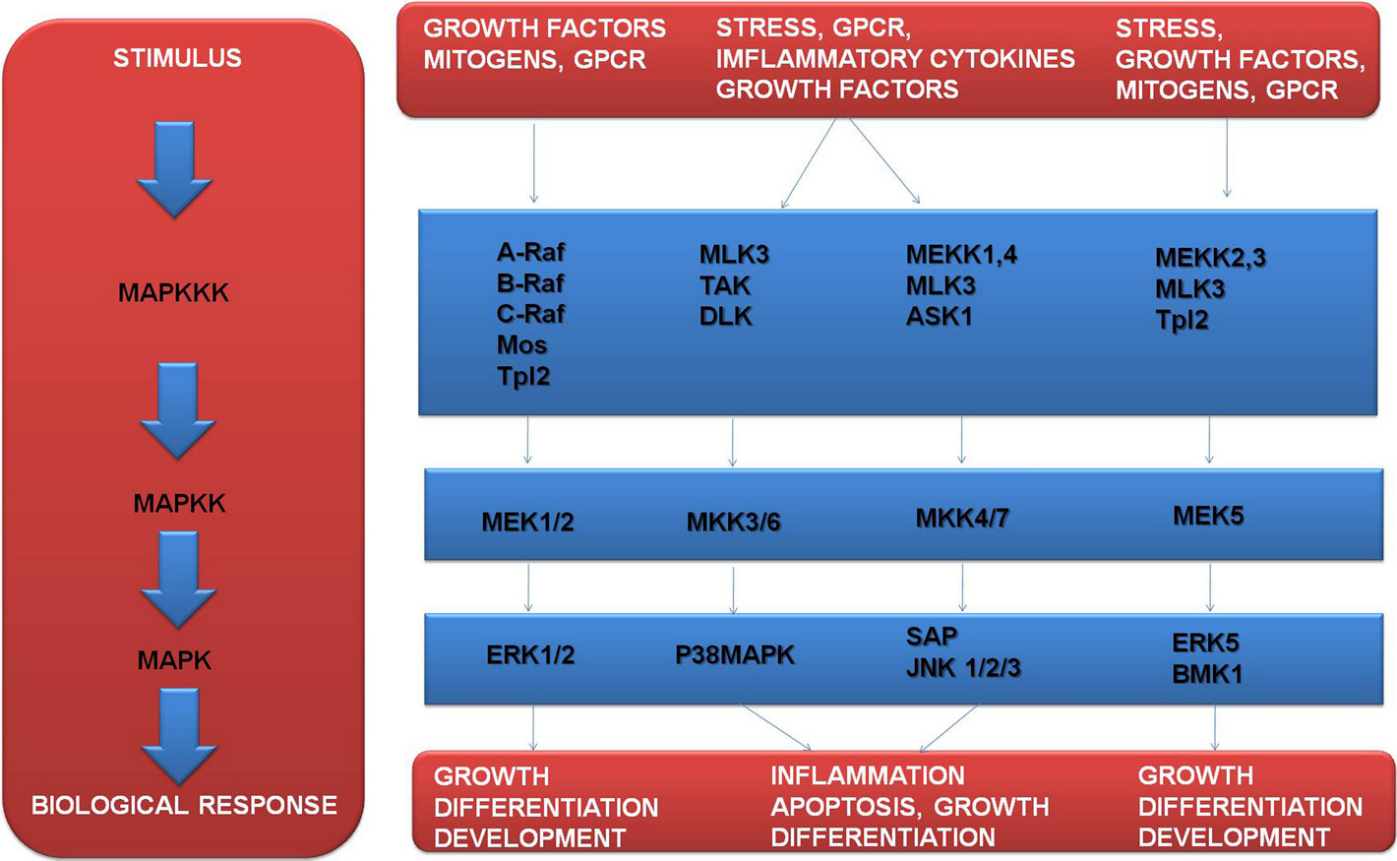
**Scheme of the three-tiered modules of the mitogen-activated protein kinases (MAPK) in eukaryotic cells.** MAPK signaling cascades are organized hierarchically into three-tiered modules (a) (b) and (c). MAPKs are phosphorylated and activated by MAPK-kinases (MAPKKs), which in turn are phosphorylated and activated by MAPKK-kinases (MAPKKKs). MAPKKKs are in turn activated by interaction with the family of small GTPases and/or other protein kinases, connecting the MAPK module to cell surface receptors or external stimuli. Phosphoproteomics strategies allow identification of several phosphorylated proteins at a given time and space, thus it is possible to study the dynamic processes of signaling networks via phosphoproteomic MS-based tools (GPCR: G-protein coupled receptor).

Important issues of eukaryotic signaling pathways [5-26] are:

When an extracellular signal is generated to induce a kinase cascade, it is often transmitted into the cell through receptors that are either kinases themselves, or through kinases associated with receptors. Often, the activation of multiple signal cascades by (a) receptors, (b) different protein PTMs, (c) crosstalk between signaling pathways and (d) feedback loops to ensure optimal signaling output and modulation (Figure 2).

(a) MAPK are a family of Ser/Thr protein kinases, which are widely conserved among eukaryotes, and are involved in many cellular programs. These programs are related to: cell proliferation, cell differentiation, cell movement, and cell death. MAPK signaling cascades are organized hierarchically into three-tiered modules. Moreover, MAPKs are phosphorylated and activated by MAPK-kinases (MAPKKs), which in turn are phosphorylated and activated by MAPKK-kinases (MAPKKKs). In addition, the MAPKKKs are in turn activated by interaction with the family of small GTPases and/or other protein kinases, connecting the MAPK module to cell surface receptors or external stimuli.

(b) The binding of receptor Tyrosine (Tyr) kinases (RTKs) to their cognate ligands at the cell surface results in receptor dimerization and autophosphorylation. Phosphorylated Tyr residues subsequently serve as docking sites to recruit signaling mediators, such as growth factor receptor-bound protein 2 (GRB2).

(c) The best studied mitogen activated protein kinases (MAPKs) are the extracellular signal regulated protein kinases (ERK). ERKs phosphorylate cytoplasmic targets that then migrate to the nucleus where they can activate transcription factors involved in cellular proliferation. ???

(d) As a general view of the orchestrated signaling pathways, it is important to know that followed by communication of the signal to different cellular compartments are (i) signal processing and (ii) amplification by plasma membrane proximal events.

(e) Multiple signaling cascades such as (i) the phosphoinositide-3 kinase (PI3K)-AKT, (ii) Ras-Raf- extracellular signal-regulated kinase (ERK) mitogen-activated protein kinase (MAPK), and (iii) signal transducer and activator of transcription (STAT) pathways are activated by the assembly of these signaling complexes.

On the other hand, (iiii) Casitas B-lineage lymphoma (CBL)-mediated ubiquitylation of RTKs controls their endocytosis and the duration of receptor signaling. In addition, binding of tumour necrosis factor-α (TNFα) to its receptor, TNFR1, induces trimerization of the receptor and recruitment of the adaptor protein TNFR1-associated death domain (TRADD).

(f) These function as a hub to assemble a multiprotein signaling complex containing TNFR-associated factor 2 (TRAF2), receptor interacting Ser/Thr protein kinase 1 (RIPK1) and nuclear factor-κB (NF-κB) essential modulator (NEMO). The result is the activation of different signaling networks, such as the ERK MAPK, p38 MAPK and NF-κB pathways. Proteins in the MAPK signaling pathways are activated by both RTKs and TNFα, which allows cells to integrate multiple signals [26-38].

Figure 2 illustrates a scheme of the three-tiered modules of the mitogen-activated protein kinases (MAPK) in eukaryotic cells.

It is important to remark that explaining complex signaling pathway phosphorylation events can be difficult. In cellular signaling pathways, a protein A phosphorylates protein B, and B phosphorylates C. However, in another signaling pathway, protein D phosphorylates A, or phosphorylates protein C. Global approaches such as phosphoproteomics, the study of phosphorylated proteins, which is a sub-branch of proteomics, combined with mass spectrometry-based proteomics, have been utilized to identify and quantify dynamic changes in phosphorylated proteins over time and space. These techniques are becoming increasingly important for the systematic analysis of complex phosphorylation networks.

## Current phosphoproteomic MS-based approaches: pros and cons of current and most used methodologies for the detection of phosphorylated proteins

### Current phosphoproteomic approaches

Several analytical techniques exist for the analysis of phosphorylation, e.g., Edman sequencing and ^32^P-phosphopeptide mapping for localization of phosphorylation sites. Unfortunately, these methods do not allow high-throughput analysis or imply very labour intense operations [39]. The advantage of using the MS tools, is that high-throughput analysis of phosphorylated protein residues can be developed [40,41]. On the other hand, phosphospecific antibodies are routinely used to immunoprecipitate and therefore to enrich in phosphorylated proteins from complex mixtures [42]. A disadvantage is that currently, there are no commercial available antibodies that are suitable for faithfully enriching all proteins that are phosphorylated. Thus, these proteins must be purified or enriched from complex mixtures using alternative methods [43].

Carrying out in-gel or in-solution trypsin digestion of protein complex mixtures [44], the resulted phosphopeptides and non-phosphopeptides can be loaded into different metal ion chromatography (e.g. Immobilized Metal ion Affinity Chromatography IMAC (Fe3^+^), and Titanium Dioxide TiO_2_[45]), in order to enrich in phosphopeptides. The enriched solution can also be submitted into different reverse-phase chromatography (e.g. Graphite powder [46], POROS R3 [43]), in order to clean and desalt those phosphopeptides previously eluted. Moreover, all these kinds of chromatographies will reduce the suppression of phosphorylated peptides in the mass spectra.

When using IMAC (Fe^3+^) and also (TiO_2_) [45], the negatively charged phosphopeptides are purified by their affinity to positively charged metal ions, but some of these methods suffer the problem of binding acidic, non-phosphorylated peptides. Ficarro and co-workers [40] circumvented this problem on IMAC (Fe^3+^) by converting acidic peptides to methyl esters. Unfortunately, methyl-esterification increases the spectra complexity and requires lyophilization of the sample, causing adsorptive losses of, in particular, phosphopeptides [46,47].

Ficarro *et al.*, [40] were able to sequence hundreds of phosphopeptides from yeast, including Slt2p kinase, but the level of phosphorylated residues identified from kinases were low compared to those from phosphoproteins highly expressed within the cell. Recently, TiO_2_ chromatography using 2,5-dihydroxybenzoic acid (DHB) was introduced as a promising strategy by Larsen *et al.,*[45]. TiO_2_/DHB resulted in a higher specificity and yield as compared to IMAC (Fe^3+^) for the selective enrichment of phosphorylated peptides from model proteins (e.g. lactoglobulin bovine, casein bovine).

Chiefly phosphopeptides from highly expressed proteins within cells can be purified, while those from phosphorylated proteins with low level expression (e.g. kinases) do not bind so well to those resins. This is due to the low proportion of this kind of protein, or on the other hand, their available amount binds to metal ions although it is not sufficient to be detected by MS. The combination of Strong Cation Exchange Chromatography (SCX) with IMAC (Fe^3+^) has been proven on yeast, resulting in a huge number of phosphorylated residues identified (over 700 including Fus3p kinase) [41]. Although more than 100 signaling proteins and functional phosphorylation sites were identified, including receptors, kinases and transcription factors, it was clear that only a fraction of the phosphoproteome was revealed [41-48].

It is clear that methodologies to enrich for phosphorylated residues from kinases should be improved. However, this is not straightforward for several reasons: (a) the low abundance of those signaling molecules within cells, (b) the stress/stimulation time-duration, as only a small fraction of phosphorylated kinases are available at any given time as a result of a stimulus. Also, (c) the time adaptation over signaling pathways is a relevant and fast factor for kinases phosphorylation, and (d) the current phosphoenrichment methods, which are mainly successful for purifying phosphopeptides from highly expressed proteins [49-51].

When peptide ions are fragmented via Collision Induced Dissociation (CID), series of y- and b- ions are formed [52,53]. The peptide sequence of these peptides is obtained by correlating mass difference between peaks in the y-ion series or between peaks in the b-ion series with amino acid residue masses.

The CID fragmentation mainly occurs on the peptide backbone, and sequence information is obtained. Related to phosphotyrosine residues, partial neutral loss is observed (HPO_3_, 80 m/z) in MS2 mode, and the phosphate group on tyrosine (Tyr) residues is more stable than on serine (Ser) and threonine (Thr) residues. Also, the phospho-finger-print characteristic of phosphotyrosine is the phosphotyrosine immonium ion (~216 Da) [54,55]. Via MS3 mode, the ion originating from neutral loss (NL) of phosphoric acid (H_3_PO_4_) can be selected for further fragmentation. Then, the selected ion is automatically selected for further fragmentation after neutral loss fragmentation. Therefore, it is possible to add extra energy for the fragmentation of peptide backbone.

Nevertheless, the MS3 mode requires that the phosphorylation on ser and thr residues be labile and conventional fragmentation via CID commonly results in the partial NL of H_3_PO_4_, (98 m/z) in MS2 mode. This is due to the gas phase β-elimination of the phosphor-ester bond and thus, dehydroalanine (Ser ~69 Da) and dehydro-2-aminobutyric acid (Thr ~83 Da) are generated [54,55].

### Alternative phosphopeptide enrichment strategies

Phosphopeptides can be de-protected and sampled under acidic conditions. BEMA (β-elimination/Michael addition) takes advantage of (a) the ease of β-elimination of phosphorylated Ser and Thr residues at basic pH, and (b) the ability to subject the resulting dehydroalanine/methyl-dehydroalanine products to Michael addition with a desired tag for affinity purification [56-58].

**Figure 3:**
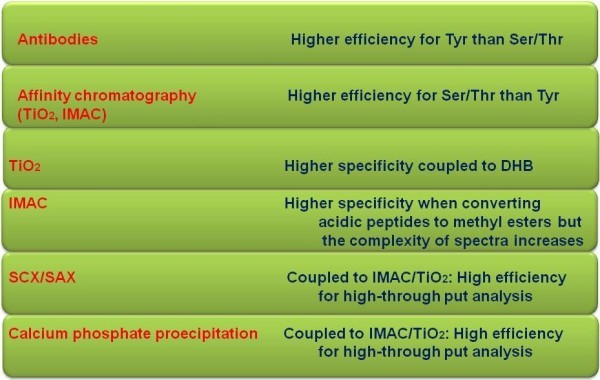
**Pros and cons of the most useful phosphoproteomic -MS based tools.** Generally, immunoprecipitation of Tyrosine via antibodies, is more efficient than antibody immunoprecipitation of Serine and Threonine residues. On the other hand, affinity chromatography of IMAC and TiO_2_, is more efficient for isolating phosphorylated residues of Serine and Threonine than Tyrosine. Moreover, TiO_2_ has shown higher specificity when it is coupled to DHB than IMAC. In addition, IMAC has shown higher specificity when converting non-phosphorylated residues (acidic peptides) to methyl esters, but the complexity of spectra increases. Furthermore, SCX, SAX and calcium phosphate precipitation methods, are more efficient when coupled to IMAC and/or TiO_2_ for clinical phosphoproteomic studies.

In addition, Calcium phosphate precipitation (CPP) has been proven to be a fast, economical, and simple enrichment technique [59] in exchange for diminished specificity. Moreover, PhosphorAmidate Chemistry (PAC) is another important approach in which phosphopeptides are coupled to a solid-phase support such as an amino-derivatized dendrimer or controlled-pore glass derivatized with maleimide for selection [60,61]. Figure 3 illustrates the most important pros and cons of several phosphoproteomic-based MS tools.

### Tandem MS methodology -basic issues- useful for phosphoproteomics via electrospray ionization (ESI)

ESI consists of a liquid-phase ion source in which the non-volatile analyte molecules are analyzed directly from the liquid phase. The development of electrospray ionization for the analysis of biological macromolecules was rewarded with the attribution of the Nobel Prize in Chemistry to John Bennett Fenn in 2002.

Firstly, the analyte molecules are diluted in an aqueous solution, and secondly this aqueous solution is forced via a fine capillary at a very low flow rate (0.1-10 μL/min).

The liquid containing the analyte(s) of interest is dispersed by electrospray into a fine aerosol. Because the ion formation involves extensive solvent evaporation, the typical solvents for electrospray ionization are prepared by mixing water with volatile organic compounds (e.g. methanol, acetonitrile).

**Figure 4:**
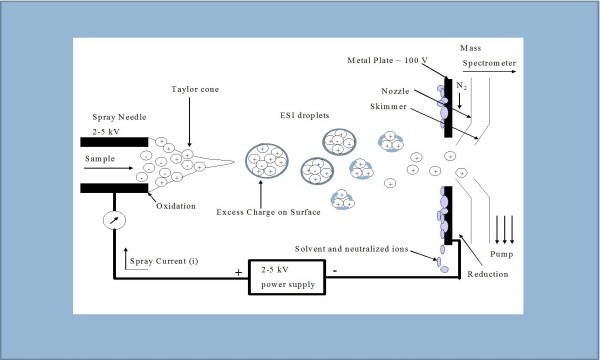
**Illustration in a simple manner of the electrospray ionization process.** The analyte solution is forced through the capillary, which has been supplied with high voltage. A Taylor cone is created due to the electric field between the capillary and the counter electrode, forming charged droplets of analyte ions and solvent. As these droplets travel towards the mass spectrometer the solvent evaporates creating analyte ions. When the solution that comprises the Taylor cone reaches the Rayleigh limit, at which point the Coulombic repulsion of the surface charge is equal to the surface tension of the solution, charged droplets are formed at the tip of the capillary. (Courtesy TE Tingholm Master Thesis 2005, PR Group, Odense Univ. Denmark).

To decrease the initial droplet size, compounds that increase the conductivity (e.g. acetic acid) are customarily added to the solution. Large-flow electrosprays can benefit from additional nebulization by an inert gas such as nitrogen. In addition, the aerosol is sampled into the first vacuum stage of a mass spectrometer through a capillary, which can be heated to aid further solvent evaporation from the charged droplets. Moreover, the solvent evaporates from a charged droplet until it becomes unstable upon reaching its Rayleigh limit. At this point, the droplet deforms and emits charged jets in a process known as Coulomb fission. After all these steps and during the fission, the droplet loses a small percentage of its mass (1.0-2.3%) along with a relatively large percentage of its charge (10-18%) [62-64]. Figure 4 illustrates the electrospray ionization process in a simple manner.

As a general rule, during MS-based experiments, a phosphopeptide mixture is separated using capillary liquid chromatography (LC). A typical separation column is 25 to 100 microns in diameter and 5 to 30 cm in length. The eluent is concurrently introduced into the mass spectrometer *via* electrospray ionization (ESI) or nanospray, which is a derivative of ESI.

ESI is a process that generates multiply protonated gas-phase peptide cations. The mass-to-charge ratio (*m/z*) and intensity (I) of the intact peptide precursors are recorded by an initial MS scan – commonly referred to as a full scan MS. Then, *m/z* values for peaks (list of masses) with high intensity are automatically selected in order of decreasing abundance for sequencing by tandem MS (MS/MS).

This process of precursor selection, dissociation, and fragment ion mass analysis is repetitively performed on analyte species as they elute from the LC column. Ideally, MS/MS interrogation of a phosphorylated peptide generates a series of fragment ions that differ in mass by a single amino acid, so that the peptide primary sequence and position of the phosphorylated modifications can be determined. This necessitates peptide bond cleavage that is not only specific to the peptide backbone, but is robust enough to elucidate differences in peptides whose primary amino acid sequences are the same, yet vary in the site of phosphorylation (e.g., positional isomers) [65].

The dominant NL peak in the fragmentation spectra of phosphopeptides obtained via traditional Collision Induced Dissociation (CID) has received much attention [66-68]. The NL peak can easily suppress sequence diagnostic ion peaks causing identification of the peptide to become very difficult and sometimes impossible.

Ion traps are the most common mass spectrometers of performing phosphoproteome analyses in CID mode. Modified fragmentation regimes have been introduced such as (a) NL triggered MS3 or (b) multistage activation (MSA), which alleviate the neutral loss issue commonly found with such instrumentation. NL MS3 and MSA methods allow fragmenting of the NL peak of the precursor ion further, in order to generate more backbone cleavages. These “extra” generated backbone cleavages, then form the more diagnostic source for peptide sequencing [69-71].

Alternatively, Electron transfer dissociation (ETD) and electron capture dissociation (ECD) have also shown great promise since the phosphate group remains attached during and after activation. Many detected phosphopeptides contain multiple Ser/Thr/Tyr residues representing the likely possibility that there is more than one possible location for the site of phosphorylation within the peptide. The abundant NL observed in low energy CID can hamper the correct assignment of the phosphor-sites in such peptides. Thus, a concerted effort has been made to understand, in detail, the rules of phosphopeptide fragmentation [72-76]. Finally, it must be said that the resulting proteomic-MS based data is pooled via bioinformatic tools, therefore, it is easier to interpret the biological and clinical meaning [77,78].

## Conclusions

(a) Phosphorylation is a key reversible modification that regulates protein function, sub-cellular localization, complex formation, degradation of proteins and therefore cell signaling networks. It can be assumed that up to 30% of all proteins may be phosphorylated, some, multiple times. Thus, it implies that phosphorylated proteins within the cell are important targets to be studied in order to improve the knowledge of many diseases.

(b) Phosphoproteomics is a branch of proteomics that identifies, catalogs, and characterizes proteins containing a phosphate group. Moreover, phosphoproteome analysis provides clues on which protein or pathway might be activated. This is due to that a change in phosphorylation status almost always reflects a change in protein activity. Indeed, it can indicate which proteins might be potential drug targets as exemplified by the kinase inhibitor. Furthermore, while phosphoproteomics will greatly expand knowledge about the numbers and types of phosphoproteins; its greatest promise is the rapid analysis of entire phosphorylation based signaling networks.

(c) Elucidating complex signaling pathway phosphorylation events can be difficult and laborious. Global approaches such as phosphoproteomics, combined with different MS strategies, have been and are being utilized to identify dynamic changes in phosphorylated proteins over time and space. These techniques are becoming increasingly important for the systematic analysis of complex phosphorylation networks, so important for clinical issues. Moreover, clinical PTMs research studies coupled to bioinformatics tools are currently and successfully being used and will elucidate relevant clues, useful for improving diagnoses and thererapies.

## Abbreviations

AQUA: Absolute Quantitation; BEMA: β-elimination/Michael addition; CID: Collision-Induced Dissociation; CPP: Calcium phosphate precipitation; Da: Dalton (molecular mass); ECD: Electron Capture Dissociation; ESI: Electron Spray Ionization; ETD: Electron Transfer Dissociation; FT-ICR: Fourier transform-Ion Cyclotron Resonance; HILIC: Hydrophilic interaction chromatography; HPLC: High-performance liquid chromatography or high-pressure liquid chromatography; H_3_PO_4_: Phosphoric acid; ICR: Ion Cyclotron Resonance; IMAC: Immobilized Metal Affinity Capture; IT: Ion Trap; iTRAQ: Isobaric Tag for Relative and Absolute Quantitation; kDa: Kilodalton (molecular mass); LC: Liquid Chromatography; MALDI: Matrix-Assisted Laser Desorption/Ionization; Mr: Relative molecular mass; MRM: Multiple reaction monitoring; MS: Mass Spectrometry; MSA: MultiStage Activation; MS/MS: Tandem mass spectrometry; m/z: Mass to charge ratio; PAC: PhosphorAmidate Chemistry; PTM: Post-Translational Modification; SILAC: Stable Isotope Labelling with Amino acid in cell Culture; SIMAC: Sequential Elution from IMAC; TiO_2_: Titanium dioxide; TOF: Time of flight.

## Competing interests

The authors declare that they have no competing interests.

## Authors’ contributions

Authors EL, XW, LM, JLP and ML carried out Functional Phosphoproteomic Mass Spectrometry-based Approaches studies for this short-review, in order to develop future Clinical Phosphoproteomic research studies and publish this article. All authors read and approved the final manuscript.
